# Feasibility and effectiveness of a two-tiered intervention involving training and a new consultation model for patients with palliative care needs in primary care: A before-after study

**DOI:** 10.1177/02692163231219682

**Published:** 2024-01-16

**Authors:** Carlos Seiça Cardoso, Filipe Prazeres, Bárbara Oliveiros, Cátia Nunes, Pedro Simões, Carolina Aires, Patrícia Rita, Joana Penetra, Paulo Lopes, Sara Alcobia, Sara Baptista, Carla Venâncio, Barbara Gomes

**Affiliations:** 1 Faculty of Medicine, University of Coimbra, Coimbra, Portugal; 2 CINTESIS@RISE, MEDCIDS, Faculty of Medicine of the University of Porto, Porto, Portugal; 3 Faculdade de Ciências da Saúde, Universidade da Beira Interior, Covilhã, Portugal; 4 USF Beira Ria, Gafanha da Nazaré, Portugal; 5 Laboratory of Biostatistics and Medical Informatics (LBIM), Faculdade de Medicina, Universidade de Coimbra, Coimbra, Portugal; 6 Coimbra Institute for Clinical and Biomedical Research (iCBR), Faculdade de Medicina, Universidade de Coimbra, Coimbra, Portugal; 7 Family Health Unit Penacova, Coimbra, Portugal; 8 Faculty of Health Sciences, University of Beira Interior, Covilhã, Portugal; 9 Personalized Health Care Unit Fundão, Fundão, Portugal; 10 Family Health Unit São Martinho de Pombal, Pombal, Portugal; 11 Personalized Health Care Unit Castanheira de Pera, Coimbra, Portugal; 12 Family Health Unit Topázio, Coimbra, Portugal; 13 Family Health Unit Rainha Santa Isabel, Torres Novas, Santarém, Portugal; 14 Family Health Unit As Gandras, Cantanhede, Coimbra, Portugal; 15 Personalized Health Care Unit Figueira-da-Foz Norte, Coimbra, Portugal; 16 Family Health Unit Condeixa, Coimbra, Portugal; 17 Cicely Saunders Institute of Palliative Care, Policy and Rehabilitation, King’s College London, London, UK

**Keywords:** Primary health care, general practitioners, palliative care, education, signs and symptoms

## Abstract

**Background:**

Evidence suggests that involving General Practitioners in the care of patients with palliative care needs may improve patient outcomes.

**Aim:**

To evaluate whether a two-tiered intervention involving training in palliative care and a new consultation model in primary care for patients with palliative care needs is feasible and could reduce patients’ symptom burden.

**Design:**

Before-after study including an internal pilot.

**Setting/participants:**

Nine general practitioners working in a health region in Portugal and 53 patients with palliative care needs from their patient lists were recruited. General Practitioners received training in palliative care and used a new primary palliative care consultation model, with medical consultations every 3 weeks for 12 weeks. The primary outcome was physical symptom burden, self-reported using the Integrated Palliative care Outcome Scale (IPOS) patient version (min.0–max.1000). Secondary outcomes included emotional symptoms (min.0–max.400) and communication/practical issues (min.0–max.300).

**Results:**

Of the 35/53 patients completed the 12-week intervention (mean age 72.53 years, SD = 13.45; 54.7% female). All had advanced disease: one third had cancer (*n* = 13), one third had congestive heart failure (*n* = 12); others had chronic kidney disease and chronic obstructive pulmonary disease. After the 12 weeks of intervention, there was a reduction in physical symptom burden [mean difference from baseline of 71.42 (95%CI 37.01–105.85) with a medium-large effect size (0.71], and in emotional symptom burden [mean difference 42.86 (95%CI 16.14–69.58), with a medium effect size (0.55)]. No difference was found for communication/practical issues.

**Conclusions:**

Our intervention can be effective in reducing patients’ physical and emotional symptoms.

**Trial registration:**

ClinicalTrials.gov ID – NCT05244590. Registration: 14th February 2022.

**What is already known on this topic?**

- The burden of chronic, progressive, incurable and life-threatening illness is increasing, highlighting the need to integrate palliative care into patients’ care plans.
- Data indicate that involving General Practitioners in the provision of palliative care may improve outcomes for patients and families, but the evidence on the effectiveness of interventions for patients with palliative care needs in primary care is still scarce.

**What this study adds?**

- We developed a training programme, from logistics to content, to be feasible for General Practitioners and to address the main topics in which they identified training needs.
- A two-tiered intervention was implemented, involving training and a new consultation model; this was shown to be feasible and effective in reducing the physical and emotional symptoms of patients with palliative needs managed in primary care.
- To the best of our knowledge, this is the first intervention involving General Practitioners, that assesses the impact on patients’ self-reported symptoms and demonstrates positive effects.

**Implications for practice, theory or policy**

- General Practitioners may test whether the intervention is applicable in their own setting, as there is potential for transferability to similar primary care settings elsewhere in the world.
- We successfully implemented and evaluated an intervention with a statistically and clinically important impact on patients, showing that research in primary palliative care can and must expand, as it may be key in the initial care of patients with palliative needs.

## Introduction

The global burden of serious health related suffering associated with progressive cancer and non-cancer conditions is estimated to nearly double by 2060.^1^ This highlights the need to integrate palliative care into health systems and into the individual care of these patients.

The World Health Organization emphasizes the inseparability of palliative care and primary health care, in guidance for planners, implementers and managers published in 2018.^2^ They suggest that primary care clinicians who have received basic training and access to a simple set of affordable and reliable medicines and equipment can effectively address the palliative care needs of most patients.^2^ It is estimated that 40 million terminally ill persons, and millions of others not imminently dying, need palliative care every year. Since primary health care is often the only available option in the community, it is both medically and morally necessary to integrate palliative care into primary care.^2^

The evidence on the effectiveness of interventions for patients with palliative care needs in primary care is still very scarce. In 2022, we conducted a systematic review of randomized clinical trials, finding only four and none demonstrated effectiveness.^3^

Given the benefits of consultations for the surveillance of some chronic diseases^4^ and the need for General Practitioners training in palliative care, we developed an intervention for patients with palliative care needs monitored in primary care, combining training and the delivery of new consultation model. Guided by the Medical Research Council framework on complex interventions,^5^ we aimed to evaluate whether this two-tiered intervention was feasible and could reduce patients’ symptom burden.

## Methods

### Study design and setting

We conducted a before-after study, incorporating an internal pilot, involving General Practitioners working in a health region in Portugal and patients with palliative care needs from their patient lists.

For context, mainland Portugal is divided into five health care regions and the Portuguese primary health care system comprises a network of health centres spread across the country. Initially designed as a pyramidal structure with top-down decision-making, it has since evolved into a decentralized system that is closer to the needs of the population. This evolution led to the creation of third-generation health centres, also known as family health units, in 1999.^6^

Our study was conducted in the centre health care region, and the nine General Practitioners who participated in the intervention worked in nine different practices in this region. The region has a population of 1.7 million people and in 2021 it had the highest aging index (228.6) of all regions of mainland Portugal (average 182.1).^7^ According to data published by the National Commission for Palliative Care in Portugal the centre health care region has the lowest number of community-based palliative care teams (two teams) out of a total of 24 teams that operate in mainland Portugal (average 4.8 teams per health care region).^8^

The study is presented following the Transparent Reporting of Evaluations with Nonrandomized Designs (TREND) statement.^9^

### Participants

#### General Practitioner eligibility criteria

General Practitioners working in the health region were considered, with no exclusion criteria. The regional health authority was asked to send an invitation to participate in the study via e-mail, with information about the study procedures. General Practitioners were informed that the number of patients that each General Practitioner would recruit would depend on the number of General Practitioners that agreed to participate in the study.

#### Patient eligibility criteria

Each General Practitioner identified patients with palliative care needs meeting the inclusion criteria (Table 1) from their list of patients following a two-step approach: first identifying all patients with neoplasm, chronic obstructive pulmonary disease (COPD), congestive heart failure (CHF) or chronic kidney disease (CKD) and then by searching patients’ medical records, identifying those with advanced disease (Supplemental File 1). Therefore, for this study, we defined a ‘patient with palliative care needs’ based on the diagnosis of metastatic cancer, COPD with GOLD classification III/IV, CHF with New York Heart Association Functional Classification (NYHA) III/IV or CKD stage IV/V.

**Table 1.** Patient eligibility criteria.

*Inclusion criteria* • Belonging to the list of patients of the recruited General Practitioners. • 18 years or older. • Diagnosis of advanced stage neoplasm – defined as metastatic neoplasm, Chronic Obstructive Pulmonary Disease (COPD) with GOLD classification III/IV, Congestive Heart Failure (CHF) with New York Heart Association Functional Classification (NYHA) III/IV, Chronic Kidney Disease (CKD) stage IV/V.
*Exclusion criteria* • Refusal, at any time, to participate in the study. • Level of understanding that compromised taking part in the study and answering the IPOS patient version^6^ (Mini Mental State Examination score <20 (moderate to severe cognitive impairment).^7^ • Level of disease severity requiring urgent intervention. This was defined according to each General Practitioner clinical judgement. Patients with a worsening of their clinical status that required prolonged hospitalization or institutionalization were thus excluded from the study.

Eligible patients were randomly ordered using a computer software and contacted by telephone (in that same random order) and invited to participate in the study. If a patient declined to participate or did not meet the inclusion criteria, the next patient on the random list was contacted; this process continued until the planned sample size was achieved. Patients’ eligibility criteria are presented in Table 1.

### Intervention

#### Training in palliative care

The General Practitioners palliative care training programme was developed based on General Practitioners needs as identified in our previous focus group study^10^ and on feedback from members of the Palliative Care Study Group of the Portuguese Association of General and Family Medicine (GEsPal). This was an e-learning programme with a 24-h load, including topics such as the role of communication, the main problems and symptoms of patients with palliative care needs and how to assess and control them (delivered by GESPal members). Additionally, the programme covered how to apply the newly developed medical consultation model and how to use the Integrated Palliative Care Outcome Scale (IPOS) patient version (programme is detailed in Supplemental File 2).

#### Consultation model

For context, Portuguese General Practitioners currently do not have any structured guidance on how to assess and manage a patient with palliative care needs during a consultation. This results in many of these patients remaining unidentified. As a result, either the patients maintain contact with their General Practitioner on their own initiative or they may go on without a defined medical follow-up. Often, healthcare contact occurs in advanced stages of the illness through hospital care, particularly via the emergency department. When this is not the case, usually contact with the General Practitioner occurs in the context of a clinical exacerbation, with symptoms in need of control. If symptom control is possible in the primary care context, then each General Practitioner manages each patient according to their training and practice. If the patient presents higher levels of complexity, then referral to a home-based or in-hospital palliative care team may be attempted, if available locally. However, patients with palliative care needs but with symptoms that are not yet severe or complex often fail to meet the referral criteria for specialist palliative care services. This results in a gap in the structured care organization for this group of patients, who remain mostly seen in the context of disease exacerbations, without a structured follow-up plan until later stages of the illness and death.

Our new primary care consultation model was developed to address this gap, being specifically designed for patients with palliative care needs based on the 4th edition of the Clinical Practice Guidelines for Quality Palliative Care of the National Coalition for Hospice and Palliative Care,^11^ as well as the core competencies of General Practitioners as defined by the World Organization of Family Doctors (WONCA) and feedback from palliative care experts in primary care. To our knowledge, there was no consultation model defined with this objective in primary care, so we met and discussed the model with General Practitioners with palliative care expertise to arrive at a periodicity and format that best met feasibility and the expected impact. We agreed on a consultation model of 12 weeks, with consultations planned every 3 weeks.

The consultation model was divided into five areas (Table 2) to be addressed by the General Practitioners gradually and regularly in their consultations throughout the 12 weeks: (1) summarizing clinical information, (2) objective symptoms’ management, (3) assessment and coding of chronic diseases and symptoms, (4) planning clinical approach and (5) addressing other problems/concerns. These five areas were constructed to be intuitive for General Practitioners and to align with the SOAP system that is currently used in the national electronic recording system for doctors to register their consultations. The SOAP system was developed by Larry Weed in 1968 to guide problem-oriented medical records, prompting doctors to record the following fields: ‘S – subjective’, ‘O – objective’, ‘A – assessment’ and ‘P – plan’.^12^ SOAP is also widely used to document care in a structured way.^13,14^

**Table 2.** Primary palliative care consultation model.

	0 weeks	3 weeks	6 weeks	9 weeks	12 weeks
1. Summarize clinical information (S)					
Main diagnosis and others clinical problems	x				
Register chronic medication and SOS	x				
Vulnerabilities (financial, housing, nutrition, security, caregiver, literacy)	x	x	x	x	x
2. Objective symptoms’ management (O)					
Recording the result of the IPOS patient version - physical symptoms	x	x	x	x	x
Other symptoms	x	x	x	x	x
Adverse medication reactions	x	x	x	x	x
3. Assessment and coding of chronic diseases and symptoms (A)					
4. Planning clinical Approach (P)					
Symptomatic treatment	x				
Review of the therapeutic record according to clinical relevance (e.g. description)	x	x	x	x	x
5. Problems/concerns (other) (P)	x	x	x	x	x

The consultation model comprises five areas that align with the SOAP system for recording medical consultations, currently in use in the Portuguese electronic medical recording system and also widely used, particularly in primary care, to document care in a structured way.^13,14^

In the first area of our consultation model (‘Summarize clinical information’ – S, Table 2), the General Practitioners assessed and registered relevant clinical and social information that could have an impact on the treatments provided, adherence to those treatments and the seeking of other social or healthcare support services tailored to each patient’s needs. General Practitioners asked and updated the list of health problems for the patient, focusing on the main diagnosis that led to their inclusion in the study, as well as other health problems. They inquired about the patient’s regular medication, both daily medications and as-needed medications. Finally, they assessed other vulnerabilities, including socio-economic status, home infrastructure, nutritional status, the presence of formal or informal caregiving and the patient’s level of education.

In the second area of the consultation model (‘Objective symptoms’ management’ – O), the General Practitioners assessed and recorded the results of the IPOS patient version. They also collected information about other relevant symptoms, registered information about physical examinations (if necessary) and inquired about potential adverse reactions to the prescribed medication. They assessed relevant symptoms and quantified their impact on the patient, in order to establish an individualized care plan.

In the third area (‘Assessment and coding of chronic diseases and symptoms’ – A) the General Practitioners updated the problem list, not only for the diagnoses previously collected but also with a view to define as active problems any symptoms identified in the previous area. This ensured a revaluation of each symptom in each consultation to assess the progression of its control.

Finally, in the fourth and fifth areas (‘Planning clinical approach’ and ‘Problems/concerns (other)’ – P), the General Practitioners developed and recorded the therapeutic plan, whether pharmacological or non-pharmacological. A review of pharmacological therapy was also conducted to assess its appropriateness, and if necessary, deprescribing was initiated. Finally, the General Practitioners addressed other patient concerns or issues, not necessarily related to the illness. Therefore, wishes, expectations and social issues were discussed and documented, so they could also be revisited in subsequent consultations.

### IPOS patient version

The IPOS patient version^15^ was used in two ways: (1) to assist General Practitioners in their assessment and intervention, guiding clinical practice, and (2) to measure study outcomes. Each General Practitioner administered the IPOS to their own patients at the beginning of the first and last consultations. This tool is a self-reported 19-item multidimensional scale built to identify the main concerns of patients in palliative care. It can be completed by the patient alone or with assistance. Item 1 is an open question regarding the three main problems or concerns that the patient had in the last week. Items 2–9 target specific symptoms and problems and are rated on 5-point Likert scales. Item 2 lists 10 of the most common physical symptoms in population with palliative care needs (pain, shortness of breath, weakness or lack of energy, nausea, vomiting, poor appetite, constipation, sore or dry mouth, drowsiness, poor mobility), with the possibility of recording three additional symptoms (not present in the list); item 3 is about anxiety; item 4 relates to family/friends concerns; item 5 asks about depression; item 6 asks if the patient ‘feels at peace’; item 7 is about sharing feelings with significant people; item 8 deals with information needs and item 9 focuses on practical problems related to the disease process. Original and translations of the IPOS and information on how to score are available at: https://pos-pal.org.

Each of the 10 physical symptoms (item 2) and all 4 emotional symptoms (items 3–6) above were linearly converted to a scale from 0 to 100, where higher scores correspond to more severe symptoms. In addition, items 7–9 were converted to a scale from 0 to 100 representing the functionality associated with each question.

The IPOS patient version has been translated and validated for the Portuguese population.^16^ Its Cronbach’s alpha showed good internal consistency (0.657) and the intraclass correlation coefficient testing reproducibility revealed very good reliability (0.794–0.950).

### Primary outcome

Our primary outcome, physical symptom burden, was obtained by summing the scores of IPOS item 2 (converted into min.0–max.1000, higher scores representing greater physical symptom burden) and comparing scores before the intervention (T0) and after 12 weeks (T12).

### Secondary outcomes

Emotional symptoms burden was obtained by summing the scores of IPOS items 3, 4, 5 and 6 emotional symptom burden (converted into min.0–max.400, higher scores meaning greater emotional symptom burden) and comparing scores between T0 and T12.

Communication/practical issues burden was obtained by summing scored of the IPOS items 7, 8 and 9 (converted into min.0–max.300, higher scores meaning greater communication/practical issues burden) and comparing scores between T0 and T12.

### Data collection

The variables about General Practitioners were collected by the first author (CSC) using a pre-defined online form and included: age, sex, years of clinical practice, type of primary care unit in which they work (Family Health Unit or Personalized Health Care Unit), environment (rural or urban) and previous training in palliative care (short training, internship, advanced training (postgraduation, masters and PhD)).

The variables bout the patients were collected by each General Practitioner using a pre-defined paper form and included: age, sex, main diagnosis and IPOS patient version scores (T0 and T12). Each form was pseudonymised by assigning an ID code, so that only each General Practitioner was able to identify which patient each form belonged to. The codebook was kept only by the General Practitioner.

### Statistical analysis

We described categorical variables using frequencies (absolute and relative), ordinal variables using median and interquartile interval and continuous variables using mean and standard deviation. We report paired-sample *T*-test results comparing T0 and T12 scores for primary and secondary outcomes. Although the scores for physical symptoms and communication/practical issues were not normally distributed (Shapiro-Wilk tests in Supplemental File 3), non-parametric tests of differences (Wilcoxon signed rank test in Supplemental File 4) produced similar results.

We have calculated effect sizes interpreted according to Cohen^17^: 0.20–0.3 as small, 0.4–0.7 as medium and ⩾0.8 as large. Statistical significance was set at *p* < 0.05.

### Sample size

A sample size of 35 patients was calculated for the primary outcome (physical symptom burden), considering the two time points (T0 and T2), a power of 80%, a type I error of 5% and a medium effect size of 0.5.^14^ To accommodate around 35% loss of patients at follow-up^18–20^ we planned to recruit 53 patients. Each General Practitioner was given an estimated recruitment target of six patients, but this was adjusted according to overall progress to reach the planned sample size.

### Pilot evaluation

We planned a pilot evaluation of the first two patients recruited by each General Practitioner, so that initial feedback could be collected and the consultation model improved accordingly. As the times for recruiting patients and first consultation were different among General Practitioners, the pilot had to be adapted and included the feedback from the first 18 patients to complete the 12-week intervention.

## Results

### Recruitment, follow-up and missing data

#### General Practitioners

**Figure 1.**
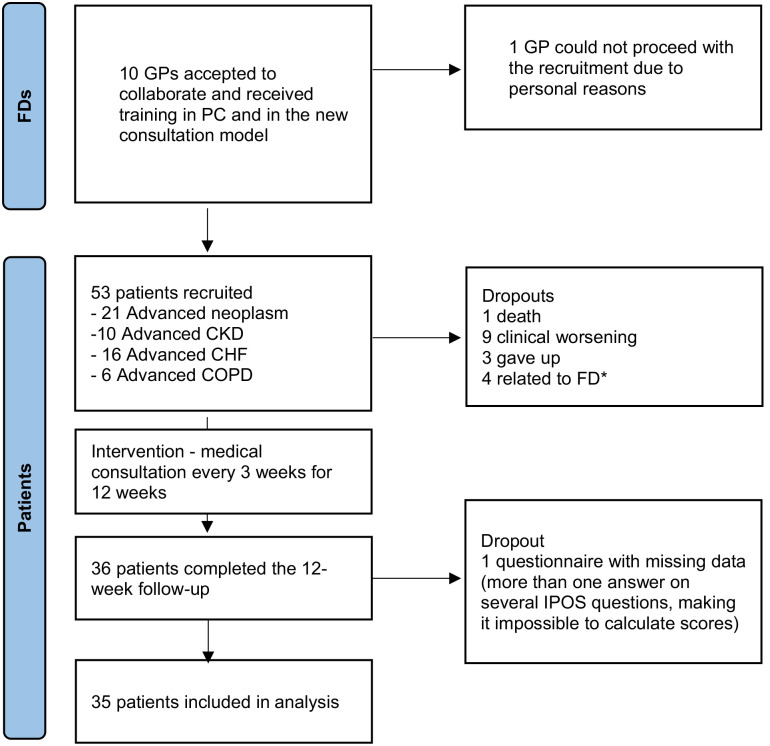
Recruitment flowchart. General Practitioners: general practitioners; PC: palliative care; CKD: chronic kidney disease; CHF: congestive heart failure; COPD: chronic obstructive pulmonary disease. *These FD recruited five patients but had extended absence due to illness, which resulted in the loss of follow-up for these four patients, with only one completing the 12th week consultation.

Ten General Practitioners agreed to participate and were enrolled in the study (flowchart in Figure 1). All received the training programme. After the programme and before starting patients’ recruitment, one General Practitioner withdrew for personal reasons, leaving nine General Practitioners to recruit patients and deliver the intervention.

The nine General Practitioners (7 female) had a mean age of 33.8 years (SD = 3.0), a mean of 3.6 years (SD = 3.2) of practice as a General Practitioner, 66.7% (*n* = 6) were working in family health units and 55.6% (*n* = 5) in a rural setting. Concerning previous training in Palliative Care, three General Practitioners indicated they had short training, one completed a 1-month internship in a home palliative care team and the others indicated they had no specific training in palliative care (Supplemental File 5).

#### Patients

The General Practitioners enrolled 53 patients who accepted to participate. They had a mean age of 72.53 years (SD = 13.5), and 54.7% were female. In terms of main diagnosis, 21 had advanced stage neoplasm, 16 had advanced CHF, 10 had advanced CKD and 6 had advanced COPD (Table 3).

**Table 3.** Patients’ characteristics.

	53 enrolled patients	35 patients for analysis
Age	Mean 72.53 (SD = 13.45)	Mean 71.97 (SD = 13.021)
Sex	45.3% male	48.6% male
	54.7% female	51.4% female
Main diagnosis		
Advanced neoplasm	39.6% (*n* = 21)	37.1% (*n* = 13)
CKD	18.9% (*n* = 19)	17.1% (*n* = 6)
CHF	30.2% (*n* = 16)	34.3% (*n* = 12)
COPD	11.3% (*n* = 6)	11.4% (*n* = 4)

CKD: chronic kidney disease; CHF: congestive heart failure; COPD: chronic obstructive pulmonary disease.

Of the 53 patients, 18 were lost to follow-up (1 died, 9 due to clinical worsening, 3 dropped out and 4 due to General Practitioner sick leave). Of the remaining 36 patients who completed the intervention, one questionnaire at T12 had invalid data and had to be excluded (more than one answer on several IPOS questions, making it impossible to calculate scores), leaving us with valid data from 35 patients for analysis (flowchart in Figure 1).

These 35 patients had a mean age of 71.97 years (SD = 13.0) and 51.4% were female. In terms of main diagnosis, 13 had Advanced Stage Neoplasm, 12 had advanced CHF, 6 had advanced CKD and 4 had advanced COPD (Table 3).

#### Pilot

In this pilot evaluation we obtained feedback from 7 General Practitioners after the first 18 patients completed the intervention. All General Practitioners expressed positive feedback, highlighting the importance of the structure of the consultation model, that periodicity gave patients safety and that the holistic approach was an important factor for doctors and patients. The main barriers identified were insufficient time for the first consultation, the difficulty of fitting patients into their consultation schedule and that, in the case of well-controlled patients, some consultations did not bring any intervention.

Given that the time for the first consultation could no longer be changed and as the definition of ‘well-controlled patients’ could be subjective, it was decided to maintain the consultation model as initially planned for the patients who had not yet completed the intervention.

On a Likert scale (0–10) evaluating the feasibility of the consultation model from the perspective of the General Practitioners, we obtained a mean score of 8 (min.6–max.10).

#### Outcomes

After 12 weeks (Table 4), there was an improvement in physical symptoms’ burden, with a mean difference from baseline of 71.42 (95%CI 37.01–105.85) and a medium-large effect size (0.71). Mean scores were at baseline 190 (SD = 147.80) and 12 weeks after 118.57 (SD = 102.24) [min.0–max.1000].

**Table 4.** Group change in patient outcomes after the intervention.

	T0	T12	Mean difference	95% CI	*p*	Effect size
Physical symptoms burden	190.00	118.57	71.43	37.01–105.85	<0.001^a^	0.71^b^
Emotional symptoms burden	139.29	96.43	42.86	16.14–69.58	0.003^a^	0.55^b^
Communication/practical issues	51.43	48.57	2.86	−21.86–25.57	0.816^a^	0.04^b^

Physical symptom burden was obtained summing the scores of IPOS item 2 (converted into min.0–max.1000, higher scores representing greater physical symptom burden) and comparing scores between T0 and T12.

Emotional symptoms burden was obtained by summing the scores of IPOS items 3, 4, 5 and 6 emotional symptom burden (converted into min.0–max.400, higher scores meaning greater emotional symptom burden) and comparing scores between T0 and T12.

Communication/practical issues burden was obtained scumming scores of the IPOS items 7, 8 and 9 (converted into min.0–max.300, higher scores meaning greater communication/practical issues burden) and comparing scores between T0 and T12.

T0: first medical consultation; T12: final medical consultation after 12 weeks.

a Related-samples *T*-test.

b Cohen’s *d*.

There was also an improvement in emotional symptoms’ burden with a mean difference of 42.86 (95%CI 16.14–69.58) and a medium effect size (0.55). Mean scores were at baseline 139.29 (SD = 91.01) and 12 weeks after 96.43 (SD = 73.55) [min.0–max.400].

No difference was found for communication/practical issues, with a mean difference of 2.857 (95%CI −21.86 to 25.57). Mean scores were at baseline 51.43 (SD = 51.79) and 12 weeks after 48.58 (SD = 58.46) [min.0–max.300].

Table 5 shows the evolution of each individual patient from baseline to 12 weeks after. Regarding physical symptoms’ burden, 25 patients (71.43%) experienced an improvement, 2 (5.71%) remained stable and 8 (22.86%) got worse. Regarding emotional symptoms’ burden, 23 patients (65.71%) experienced improvement, 7 (20.00%) remained stable and 5 (14.29.86%) got worse. Regarding communication/practical issues, 17 patients (48.57%) experienced improvement, 8 (22.86%) remained stable and 10 (28.57%) got worse.

**Table 5.** Individual change in patient outcomes after the intervention.

Patient	Physical symptoms’ burden		Emotional symptoms’ burden		Communication/practical issues	
	T0	T12	T0	T12	T0	T12
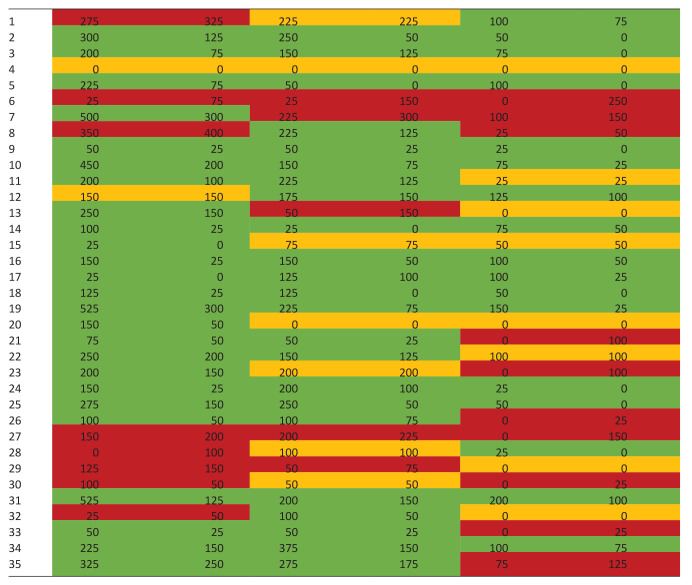						

The values highlighted in green represent the scores that improved between T0 and T12, those highlighted in yellow represent those who remained unchanged and those highlighted in red represent the scores that have worsened.

## Discussion

### Main findings

The intervention has shown to be feasible and effective in reducing the physical and emotional symptoms of patients with palliative needs managed in primary care.

### What this study adds?

Despite the limitations inherent to a before-after study design, the findings demonstrate a promising role for General Practitioners in the care of patients with palliative care needs. To the best of our knowledge, this is the first intervention involving General Practitioners that assesses the impact on patients’ self-reported symptoms, and demonstrating positive effects.

Furthermore, the tools developed for this study, including the training and consultation model, can be well-suited for other General Practitioners and may hold value in transferring this intervention to other primary care settings worldwide.

### Strengths and limitations

Another strength of the intervention is the procedures of development of the training programme for General Practitioners. While there are several courses and advanced training programmes available, they may not always be designed to meet the expectations of the professionals who will receive them. This programme was designed, from logistics to content, to be feasible for General Practitioners and to address the main topics where they identified training needs. Given the World Health Organization call for primary care clinicians to have received basic palliative care training,^2^ and considering advocacy for training in palliative care to be mandatory during General Practitioners’ internships,^21^ our training programme could be helpful in achieving that, if extended beyond the study.

There are, however, some limitations. We used diagnosis without a standard tool to identify patients with palliative care needs. With the exception of the surprise question (with low sensitivity [63.9%] and specificity [63.7%]),^22^ we found no tool to identify palliative care needs validated for the Portuguese population (research gap). Although the application of such tool could be desirable, it would have reduced our sample, potentially excluding less symptomatic patients.

This may help explain why the baseline level of symptoms was not high, which may limit the extrapolation of results to patients with higher symptom burden. Nevertheless, the purpose of the study and of the intervention was to promote the introduction of palliative care earlier, allowing the patient to benefit for a longer time. This aligns with evidence from other care settings, showing earlier palliative care is better for patients’ quality of life and may even improve their survival.^23–28^ Allowing for intervention in earlier/less symptomatic stages of the disease makes it possible for professionals who are not palliative care experts to be involved in delivering palliative care, in this case primary palliative care.

The age and years of experience of the General Practitioners were relatively low. In our previous focus group,^10^ the participating General Practitioners were also generally young. This may indicate a greater interest among younger generations in this field. While this demographic factor should be considered when appraising the study results, it also presents a window of opportunity and hope for the growth of primary palliative care in the coming years. The enthusiasm and interest of younger General Practitioners could lead to the continued development and expansion of further initiatives in the future.

A major limitation of this study is the lack of a control group and a randomized clinical trial design, which would reduce bias and provide more definite results. Still, our results demonstrate the feasibility and show that, with the intervention, symptoms reduced in 12 weeks, contrary to what would be expected with disease progression. The effect size we found in our primary outcome (0.71—medium–large) is higher than what we expected (0.5) and uncommon both in primary and palliative care research.^29^ This supports a methodological step-up, which we will do in the near future. In the meantime, General Practitioners may test if the intervention is applicable in their own setting, as there is potential for transferability to similar primary care settings elsewhere in the world.

## Conclusion

Our two-tiered intervention has proved to be feasible and can be effective in reducing the symptoms of patients with palliative needs in primary care, delivering palliative care for more.

## Supplemental Material

sj-docx-1-pmj-10.1177_02692163231219682 – Supplemental material for Feasibility and effectiveness of a two-tiered intervention involving training and a new consultation model for patients with palliative care needs in primary care: A before-after study Supplemental material, sj-docx-1-pmj-10.1177_02692163231219682 for Feasibility and effectiveness of a two-tiered intervention involving training and a new consultation model for patients with palliative care needs in primary care: A before-after study by Carlos Seiça Cardoso, Filipe Prazeres, Bárbara Oliveiros, Cátia Nunes, Pedro Simões, Carolina Aires, Patrícia Rita, Joana Penetra, Paulo Lopes, Sara Alcobia, Sara Baptista, Carla Venâncio and Barbara Gomes in Palliative Medicine

sj-docx-2-pmj-10.1177_02692163231219682 – Supplemental material for Feasibility and effectiveness of a two-tiered intervention involving training and a new consultation model for patients with palliative care needs in primary care: A before-after study Supplemental material, sj-docx-2-pmj-10.1177_02692163231219682 for Feasibility and effectiveness of a two-tiered intervention involving training and a new consultation model for patients with palliative care needs in primary care: A before-after study by Carlos Seiça Cardoso, Filipe Prazeres, Bárbara Oliveiros, Cátia Nunes, Pedro Simões, Carolina Aires, Patrícia Rita, Joana Penetra, Paulo Lopes, Sara Alcobia, Sara Baptista, Carla Venâncio and Barbara Gomes in Palliative Medicine

sj-docx-3-pmj-10.1177_02692163231219682 – Supplemental material for Feasibility and effectiveness of a two-tiered intervention involving training and a new consultation model for patients with palliative care needs in primary care: A before-after study Supplemental material, sj-docx-3-pmj-10.1177_02692163231219682 for Feasibility and effectiveness of a two-tiered intervention involving training and a new consultation model for patients with palliative care needs in primary care: A before-after study by Carlos Seiça Cardoso, Filipe Prazeres, Bárbara Oliveiros, Cátia Nunes, Pedro Simões, Carolina Aires, Patrícia Rita, Joana Penetra, Paulo Lopes, Sara Alcobia, Sara Baptista, Carla Venâncio and Barbara Gomes in Palliative Medicine

sj-docx-4-pmj-10.1177_02692163231219682 – Supplemental material for Feasibility and effectiveness of a two-tiered intervention involving training and a new consultation model for patients with palliative care needs in primary care: A before-after study Supplemental material, sj-docx-4-pmj-10.1177_02692163231219682 for Feasibility and effectiveness of a two-tiered intervention involving training and a new consultation model for patients with palliative care needs in primary care: A before-after study by Carlos Seiça Cardoso, Filipe Prazeres, Bárbara Oliveiros, Cátia Nunes, Pedro Simões, Carolina Aires, Patrícia Rita, Joana Penetra, Paulo Lopes, Sara Alcobia, Sara Baptista, Carla Venâncio and Barbara Gomes in Palliative Medicine

sj-docx-5-pmj-10.1177_02692163231219682 – Supplemental material for Feasibility and effectiveness of a two-tiered intervention involving training and a new consultation model for patients with palliative care needs in primary care: A before-after study Supplemental material, sj-docx-5-pmj-10.1177_02692163231219682 for Feasibility and effectiveness of a two-tiered intervention involving training and a new consultation model for patients with palliative care needs in primary care: A before-after study by Carlos Seiça Cardoso, Filipe Prazeres, Bárbara Oliveiros, Cátia Nunes, Pedro Simões, Carolina Aires, Patrícia Rita, Joana Penetra, Paulo Lopes, Sara Alcobia, Sara Baptista, Carla Venâncio and Barbara Gomes in Palliative Medicine
