# Supplementary material for: Feasibility and effectiveness of a two-tiered intervention involving training and a new consultation model for patients with palliative care needs in primary care: A before-after study
Source: Palliat Med. 2024 Jan 16;38(8):842–52. doi: 10.1177/02692163231219682 (PMC11445974; doi:10.1177/02692163231219682)
Supplement: sj-docx-1-pmj-10.1177_02692163231219682 – Supplemental material for Feasibility and effectiveness of a two-tiered intervention involving training and a new consultation model for patients with palliative care needs in primary care: A before-after study [file sj-docx-1-pmj-10.1177_02692163231219682.docx]

Supplementary file 1

**Patients’ selection and allocation procedures**

Patients meeting the inclusion criteria were identified using a two-step approach. At first, all patients with eligible main diagnoses were identified (neoplasm, COPD, CHF and CKD), regardless of the severity status. To get this information, each General Practitioner ran the network MIM@UF, a national clinical repository that integrates all codification data from General Practitioners. Each General Practitioners collaborating identified patients belonging to their own patient list. In the MIM@UF, the classification of patient problems and diseases is made with the International Classification of Primary Care, 2^nd^ edition (ICPC-2).

After identifying all patients with the diagnoses that fit study criteria, each General Practitioner screened the clinical records to identify those meeting the severity criteria (ASN, COPD Gold III/IV, CHF NYHA III/IV and CKD stage IV/V). To access this information, General Practitioners evaluated the consultation records, notes associated with the coding of each diagnosis (the program used for General Practitioners records in Portugal allows associating comments in free text to a diagnosis, being possible that these notes can translate the degree of severity of the disease) and evaluate the patients’ exams recorded that allow to define the severity of each diagnosis.

Eligible patients from each General Practitioner list were then ordered randomly. Following that random order, patients were contacted by phone-call and invited to participate in the study. Those who showed interest in participating were asked to give their written informed consent. Whenever a patient meets any of the exclusion criteria, the General Practitioner move forward to the next patient of the list, repeating this procedure until the planned sample was achieved.
