# Supplementary material for: Feasibility and effectiveness of a two-tiered intervention involving training and a new consultation model for patients with palliative care needs in primary care: A before-after study
Source: Palliat Med. 2024 Jan 16;38(8):842–52. doi: 10.1177/02692163231219682 (PMC11445974; doi:10.1177/02692163231219682)
Supplement: sj-docx-2-pmj-10.1177_02692163231219682 – Supplemental material for Feasibility and effectiveness of a two-tiered intervention involving training and a new consultation model for patients with palliative care needs in primary care: A before-after study [file sj-docx-2-pmj-10.1177_02692163231219682.docx]

Supplementary file 2

**General Practitioners’ training in palliative care**

The palliative care training program for General Practitioners met the main training needs of General Practitioners according to our previous study,^7^ and feedback from elements of the Palliative Care Study Group of the Portuguese Association of General and Family Medicine (GesPal). The training program had a total duration of 24 hours, e-learning, and consisted of two modules.

The first module was taught by the elements of the GesPal and addressed the role of General Practitioners in palliative care, the role of communication, the main problems and symptoms of patients with palliative care and how to assess and control them.

The second module was taught by the first author (CSC, a General Practitioner with palliative care training) and addressed the consultation model, how to apply it in the context of a primary care medical consultation and how to apply the IPOS patient version integrated in routine clinical practice.

Thus, the course program was, in detail:

Day1: principles of palliative care (2 hours), principles of symptomatic control and symptoms other than pain (6 hours)

Day 2: communication (4 hours), pain management (4 hours)

Day 3: symptoms other than pain (4 hours), situation of last hours or days of life (2 hours), consultation template (2 hours).
