# Supplementary material for: Feasibility and effectiveness of a two-tiered intervention involving training and a new consultation model for patients with palliative care needs in primary care: A before-after study
Source: Palliat Med. 2024 Jan 16;38(8):842–52. doi: 10.1177/02692163231219682 (PMC11445974; doi:10.1177/02692163231219682)
Supplement: sj-docx-4-pmj-10.1177_02692163231219682 – Supplemental material for Feasibility and effectiveness of a two-tiered intervention involving training and a new consultation model for patients with palliative care needs in primary care: A before-after study [file sj-docx-4-pmj-10.1177_02692163231219682.docx]

Supplementary file 4

Wilcoxon signed rank test for variables without normal distribution.

Z – Wilcoxon signed rank tests’ statistic
