# Supplementary material for: Feasibility and effectiveness of a two-tiered intervention involving training and a new consultation model for patients with palliative care needs in primary care: A before-after study
Source: Palliat Med. 2024 Jan 16;38(8):842–52. doi: 10.1177/02692163231219682 (PMC11445974; doi:10.1177/02692163231219682)
Supplement: sj-docx-5-pmj-10.1177_02692163231219682 – Supplemental material for Feasibility and effectiveness of a two-tiered intervention involving training and a new consultation model for patients with palliative care needs in primary care: A before-after study [file sj-docx-5-pmj-10.1177_02692163231219682.docx]

Supplementary file 5

General Practitioners’ characteristics

|  | **Mean (sd)** |
| --- | --- |
| Age | 33.78y (3.03) |
| Years of practice as GP | 3.56y (3.17) |
|  |  |
|  | N (%) |
| Sex | 2 (22.22%) male  7 (77.89%) female |
| Type of unit | 6 (66.67%) FHU^1^  3 (33.33%) HCU^1^ |
| Work setting | 5 (55.56%) rural setting  4 (44.44%) urban setting |
| Previous training in palliative care | 3 (33.33%) short training  1 (11.11%) internship*  5 (55.56%) no specific training |

GP – General Practitioner, FHU – Family health unit, HCU - Health care units, PC – palliative care

1 – FHU and HCU are the two types of primary care units in Portugal. They differ mainly in the organization structure, with the FHU having greater organizational autonomy when compared to HCU.

* Internship of 1 month in a home palliative care team
